# Unveiling the immune microenvironment in diabetic nephropathy: from mechanisms to therapeutics

**DOI:** 10.3389/fimmu.2026.1750484

**Published:** 2026-03-06

**Authors:** Tao Jiang, Ran Chen, Jianying Chang, Jiajia Duan, Yanqiu Wang, Yuhan Sun, Xiaowan Chen, Yujin Ma, Liwen Zhang, Hongwei Jiang, Hetao Chen

**Affiliations:** 1 Luoyang Key Laboratory of Transplantation And Immunological Studies for Haematological Diseases, Department of Clinical Laboratory, The First Affiliated Hospital, and College of Clinical Medicine of Henan University of Science and Technology, Luoyang, China; 2 Luoyang Key Laboratory of Clinical Multi-omics and Translational Medicine, Key Laboratory of Hereditary Rare Diseases of Health Commission of Henan Province, Henan Key Laboratory of Rare Diseases, Endocrinology and Metabolism Center, The First Affiliated Hospital, and College of Clinical Medicine of Henan University of Science and Technology, Luoyang, China

**Keywords:** diabetic nephropathy, fibrosis, gut-kidney axis, immune microenvironment, macrophage polarization

## Abstract

Diabetic nephropathy (DN) is one of the most common microvascular complications of diabetes and is the primary cause of end-stage renal disease, imposing a significant burden on patients’ health, the medical and economic systems. The traditional view holds that the pathogenesis of DN is mainly related to metabolic disorders caused by hyperglycemia, genetic susceptibility, and abnormal hemorheology. However, with the deep integration of immunology and nephrology research, more and more evidence indicates that immune factors play a core role in its pathogenesis. The renal immune microenvironment is a network composed of immune cells, cytokines, and matrix components, which promotes the progression of the disease through continuous inflammation, immune cell infiltration, and imbalanced homeostasis. This study systematically reviews the core mechanisms of the immune microenvironment in the occurrence and development of DN, focusing on the interaction between immune cells and innate renal cells, as well as the regulatory roles of the intestinal microbiota and immune axes. We also summarize possible diagnostic markers and treatment strategies related to immunity, aiming to provide new ideas for precision medicine of DN.

## 1 Introduction

DN is a chronic kidney disease characterized by progressive renal structural damage and a persistent decline in renal function. As a major complication of diabetes mellitus, DN has emerged as an increasingly pressing global health concern (1). Approximately 30% to 40% of individuals with diabetes worldwide will develop DN, which is now the leading cause of end-stage renal disease (ESRD) (2). Diabetic nephropathy is one of the main causes of end-stage renal disease. Once patients reach the end-stage, they need to rely on dialysis or kidney transplantation to sustain their lives. This not only severely impairs the quality of life of the patients, but also imposes a huge economic and resource burden on the global healthcare system (2, 3). Therefore, analyzing its pathogenesis and developing effective intervention strategies carry substantial clinical significance. Traditional studies have primarily focused on direct renal damage resulting from metabolic disorders. However, emerging evidence has established that dysregulation of the immune microenvironment serves as a critical link between metabolic abnormalities and renal pathological injury. The homeostasis of the renal immune microenvironment is disrupted, manifested by abnormal infiltration of immune cells, excessive production of inflammatory cytokines, and dysfunction between immune cells and the native cells of the kidney. This disruption is not only a characteristic pathological feature of diabetic nephropathy but also a key factor driving the disease from early proteinuria to end-stage renal failure (4).In-depth exploration of the dynamic changes in the immune microenvironment of diabetic nephropathy can not only clarify the “metabolism-immunity-renal injury” regulatory axis to improve the pathological and physiological theories, but also provide key targets for the development of early diagnostic markers and precise therapies, thereby helping to achieve the clinical goals of “early detection, early intervention, and delay progression” (5). Currently, therapeutic interventions targeting the immune microenvironment have emerged as a research focus in diabetic nephropathy. Approaches such as modulating macrophage polarization, restoring Th17/Treg cell balance, and inhibiting the NF-κB and NLR family pyrin domain-containing 3 (NLRP3) inflammasome signaling pathways have been validated in animal models to effectively attenuate renal inflammation and fibrosis (6, 7). Inhibitors targeting Interleukin-18 (IL-18) and transforming growth factor-β (TGF-β) have entered the clinical trial stage, and some of these drugs have demonstrated favorable renal protective effects (8, 9). Furthermore, as key regulators of the immune microenvironment, the gut microbiota and its metabolites—such as short-chain fatty acids (SCFAs) and trimethylamine N-oxide (TMAO)—have been shown to modulate renal immune homeostasis, thereby providing novel “gut-kidney axis” targets for disease intervention (10). The present review comprehensively summarizes the research progress on the immune microenvironment in diabetic nephropathy from four perspectives: the composition of the immune microenvironment, its mechanisms of action, diagnostic biomarkers, and therapeutic strategies, with the aim of providing a reference for future research and clinical translation.

## 2 Composition of the immune microenvironment

**Figure 1:**
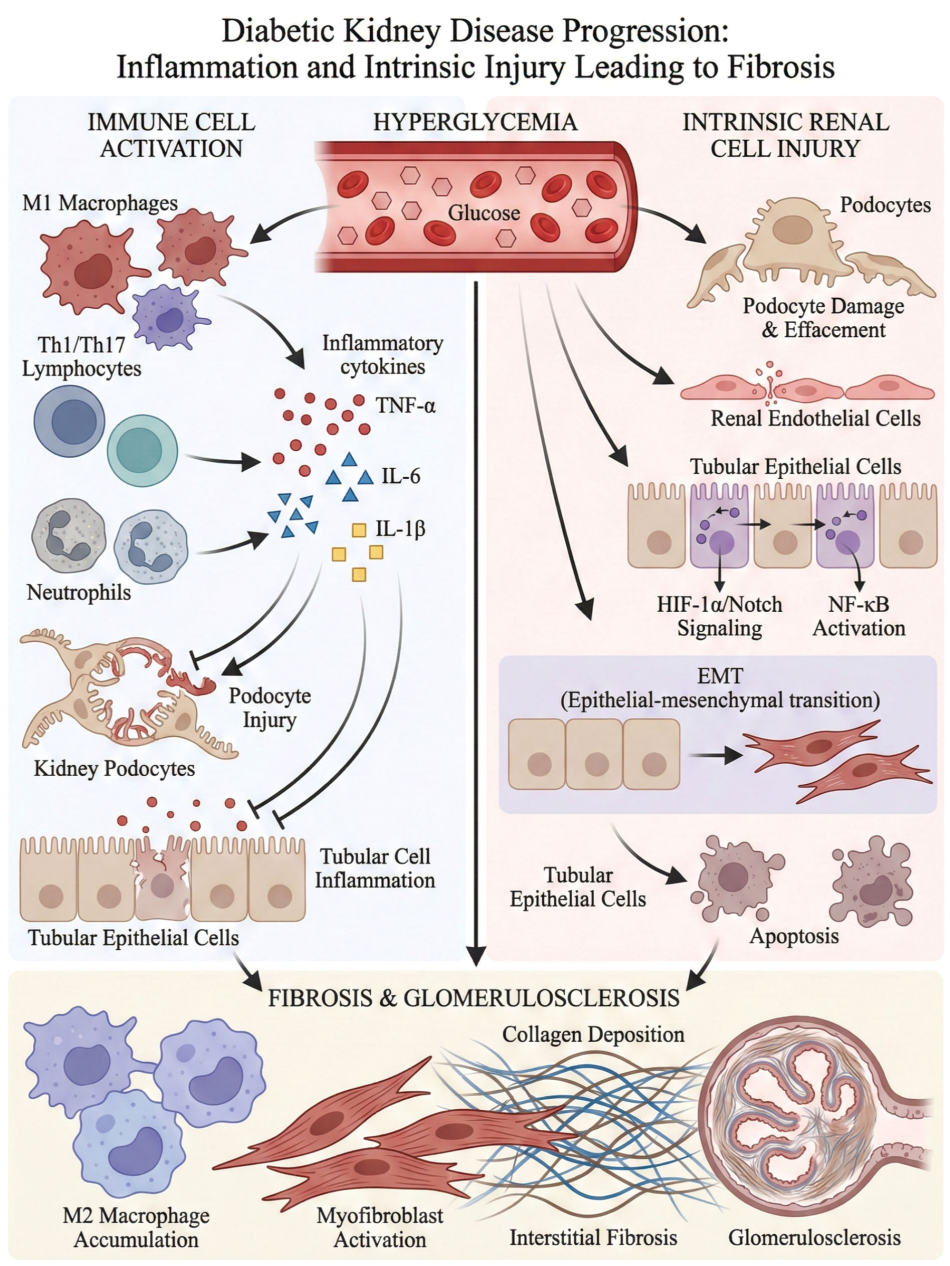
Diabetic kidney disease progression inflammation and intrinsic injury leading to fibrosis. During the development of diabetic nephropathy, persistent high blood sugar triggers systemic and local inflammatory responses in the kidneys, activating various immune cells such as M1-type macrophages, Th1/Th17 lymphocytes, etc., and releasing inflammatory factors such as TNF-α, IL-6, and IL-1β. These inflammatory mediators subsequently cause damage to the intrinsic cells of the kidneys (including podocytes, renal endothelial cells, and renal tubular epithelial cells), activate key signaling pathways such as HIF-1α/Notch and NF-κB, and induce epithelial-mesenchymal transition (EMT) and cell apoptosis. This continuous damage and imbalance in repair eventually promote kidney fibrosis and glomerular sclerosis, manifested as collagen deposition, activation of myofibroblasts, interstitial fibrosis, and sclerosis of the glomerular structure, leading to progressive loss of renal function.

The immune microenvironment consists of immune cells, non-immune cells, cytokines, chemokines, and the Extracellular Matrix (ECM). Under normal physiological conditions, this environment maintains a state of relative homeostasis. However, in the DN, factors such as hyperglycemia, advanced glycation end products(AGEs), and oxidative stress disrupt this equilibrium, resulting in functional impairments of immune microenvironment components and dysregulated intercellular communication (11). Diabetic Kidney Disease: Inflammation, Cell Injury, and Fibrotic Progression (**Figure 1**).

### 2.1 Immune cells

Immune cells mainly comprise macrophages, T cells, B cells, dendritic cells (DCs), and neutrophils. In DN, the infiltration and activation of these immune cells represent a critical mechanism underlying renal inflammation and fibrosis (12).

#### 2.1.1 Macrophages

Macrophages, as multifunctional cells of the innate immune system, can promote inflammation through the release of pro-inflammatory factors while also alleviating it via anti-inflammatory secretions (13). The inflammatory mediators it secretes can stimulate the increase of ECM production in kidney cells and reduce its degradation, thereby directly aggravating renal fibrosis. At the same time, macrophages also cause podocyte apoptosis and kidney damage through various mechanisms such as producing cytokines, regulating intracellular signaling pathways, inducing oxidative stress, and directly interacting with podocytes. Through these interrelated pathways, macrophages play a crucial role in the initiation and progression of DN (14). They play a crucial role in DN, as they are consistently present in the glomeruli and renal interstitium across all stages of the disease (15). From the perspective of disease progression, in the early stage of DN, factors such as high blood sugar and AGEs can activate the intrinsic cells of the kidneys. These activated cells subsequently upregulate the expression of various chemokines and adhesion molecules, such as MCP-1, ICAM-1, and VCAM-1, thereby recruiting and guiding circulating monocytes to infiltrate the renal tissue. The infiltrated monocytes differentiate into M1 macrophages in the renal microenvironment, resulting in an imbalance in the M1/M2 phenotypic ratio within the kidney and reaching a peak. The dominant M1 macrophages release a large amount of pro-inflammatory cytokines, triggering early inflammatory responses in the glomeruli and renal tubulointerstitial tissues. Eventually, this leads to mesangial cell proliferation, podocyte damage, and microalbuminuria (16, 17). In the middle stage of DN, evidence from animal models and human renal biopsies indicates that macrophage infiltration in the glomeruli peaks, with M1-type macrophages remaining predominant. These M1 macrophages contribute to renal injury through the production of cytotoxic mediators such as reactive oxygen species (ROS) and nitric oxide (NO). Concurrently, they secrete pro-fibrotic factors—including TGF-β and platelet-derived growth factor (PDGF)—which stimulate mesangial cells to overproduce ECM, thereby accelerating glomerulosclerosis (18). In the late stage of DN, the proportion of M2 macrophages increases markedly, leading to a decline in the M1/M2 ratio to its lowest point. Although M2 macrophages exert anti-inflammatory effects through the secretion of interleukin-10 (IL-10), their pro-fibrotic activity becomes predominant during this phase. M2 macrophages not only differentiate into myofibroblasts and directly contribute to ECM deposition but also secrete fibroblast growth factor (FGF) and connective tissue growth factor (CTGF), thereby accelerating tubulointerstitial fibrosis and ultimately resulting in irreversible renal dysfunction (19). Furthermore, renal macrophages can be categorized into two distinct subtypes: resident and infiltrating macrophages. Resident macrophages are activated early in the disease process through recognition of damage-associated molecular patterns (DAMPs), thereby initiating local inflammatory responses and recruiting circulating monocytes. DAMPs are endogenous molecules released from damaged or dead cells that are recognized by the innate immune system. This recognition triggers innate immune responses and promotes inflammation. Infiltrating macrophages progressively accumulate in the kidneys during disease progression, amplifying inflammation, inducing cellular apoptosis, and promoting fibrotic remodeling (20).

#### 2.1.2 T cells

As an important component of the adaptive immune system, T cells display remarkable heterogeneity in phenotype and function. They participate in the occurrence and development of DN by regulating inflammatory responses, mediating cytotoxic effects, and maintaining immune balance. Classified by their surface markers and functional characteristics, T cells are mainly divided into CD4^+^T cells, CD8^+^T cells, and regulatory T cells (Treg). The dynamic imbalance among these subsets is a typical feature of immune dysregulation in DN. Among them, CD4^+^ and CD8^+^T cells, as key effector cells, directly or indirectly recruit and activate other immune cells by secreting pro-inflammatory cytokines, thereby exacerbating tubulointerstitial injury and amplifying the inflammatory cascade reaction (21). CD4^+^T cells can differentiate into multiple subsets with distinct functions, and their classification is based on the cytokines they secrete. T helper 1 (Th1) cells mainly produce pro-inflammatory cytokines, such as interferon-γ (IFN-γ) and tumor necrosis factor-α (TNF-α), which drive delayed-type hypersensitivity reactions and activate macrophages to enhance their phagocytic and cytotoxic functions, thereby exacerbating local renal inflammation. In DN, the proportion of Th1 cells is elevated. The IFN-γ they secrete can induce mesangial cells to express chemokines including CXCL9 and CXCL10, thereby promoting the infiltration of more immune cells. Furthermore, IFN-γ can induce apoptosis in renal tubular epithelial cells, accelerating the progression of renal dysfunction (21).T helper 2 (Th2) cells are characterized by the secretion of Interleukin-4 (IL-4), Interleukin-5 (IL-5) and Interleukin-10 (IL-10). Among these cytokines, IL-4 and IL-5 inhibit Th1 cell activity, promote immunoglobulin E (IgE) antibody production and regulate humoral immunity, while IL-10 exerts a broad anti-inflammatory effect. In DN, a reduced proportion of Th2 cells induces a Th1/Th2 imbalance, which leads to impaired anti-inflammatory capacity and exacerbated renal inflammatory responses (22). T helper 17 (Th17) cells, through the secretion of pro-inflammatory cytokines such as interleukin-17A (IL-17A) and interleukin-17F (IL-17F), serve as a critical link between innate and adaptive immunity. In DN, hyperglycemia promotes Th17 cell differentiation via activation of the STAT3 signaling pathway.IL-17A produced by these cells induces the production of inflammatory mediators, including IL-6 and CXCL8, in intrinsic renal cells, such as glomerular mesangial cells and renal tubular epithelial cells, and further triggers neutrophil infiltration. Concurrently, IL-17A directly stimulates fibroblasts to synthesize collagen, thereby promoting renal interstitial fibrosis (23, 24). Treg cells maintain immune homeostasis by secreting anti-inflammatory cytokines such as IL-10 and TGF-β, which suppress the activation and proliferation of Th1 and Th17 cells. In DN, hyperglycemia-induced oxidative stress impairs Treg cell function, resulting in reduced cell numbers and diminished secretion of anti-inflammatory cytokines, thereby exacerbating the Th17/Treg imbalance. This dysfunction not only amplifies renal inflammation but also compromises the regenerative capacity of renal tissues, accelerating disease progression (25). CD8^+^T cells, also known as cytotoxic T lymphocytes (CTLs), recognize antigens presented by major histocompatibility complex (MHC) class I molecules. They eliminate infected or malignant target cells by releasing cytotoxic mediators such as perforin and granzyme B, and thus play a central role in cellular immune defense. In DN, CD8^+^T cells are markedly increased in both glomerular and tubulointerstitial regions. These infiltrating cells secrete pro-inflammatory cytokines, including IFN-γ and TNF-α, which promote macrophage activation and contribute to a self-sustaining “inflammatory amplification cycle”. Furthermore, CD8^+^T cells directly induce injury to podocytes and renal tubular epithelial cells, leading to disruption of renal architecture and impairment of kidney function (26).

#### 2.1.3 B Cells

B cells mediate immune regulation in the progression of DN via their functional duality. Pathogenic B−cell subsets exacerbate renal injury by secreting pro−inflammatory factors, releasing cytotoxic granules, and migrating to sites of renal inflammation. Conversely, protective B−cell subsets and related molecules suppress chronic kidney inflammation and maintain immune homeostasis, thereby conferring disease protection. Elevated levels of these protective components are significantly associated with a reduced risk of diabetic nephropathy (27). Hyperglycemic microenvironment triggers the activation of the nuclear factor-κB (NF-κB) signaling pathway in B cells, thus driving excessive secretion of pro-inflammatory cytokines and enhancing their antigen-presenting capacity. This further activates T cells and other immune cells in the body, ultimately leading to a vicious cycle of immune dysregulation (28). As professional antigen-presenting cells (APCs), B cells possess the ability to capture and process renal autoantigens. Subsequently, they present the processed antigens to CD4^+^ T cells through MHC class II molecules. This specific interaction activates Th1, Th17 and other pathogenic T cell subsets, which further amplifies renal immune responses. In type 1 diabetic nephropathy (T1DN), the role of B cells in presenting pancreatic islet autoantigens is well established. In type 2 diabetic nephropathy (T2DN), the presentation of renal autoantigens by B cells may constitute a key mechanism contributing to disease progression (29). Persistent hyperglycemia induces abnormal glycosylation in the renal tissue cells, thereby generating novel self-antigenic epitopes. These antigens trigger the differentiation of B cells into plasma cells, which are responsible for producing specific autoantibodies. The formed immune complexes then deposit in the glomerular basement membrane or mesangial regions. Such deposition not only directly impairs the glomerular filtration barrier, but also activates the complement pathways of complement component 3 (C3) and complement component 5 (C5). This activation further leads to the release of C5a—a potent chemotactic factor that promotes inflammatory cell infiltration and exacerbates renal injury (30). During the early phase of DN, the infiltration of B cells, T cells, and dendritic cells in the kidneys exhibits a significant correlation with glomerular dilation and proteinuria. This indicates that B cells play a crucial role in the immune initiation during the early stage of the disease (31). The core pathogenic mechanism underlying this B cell-mediated early renal injury is associated with the secretion of pro-inflammatory cytokines such as IL-17. This cytokine enhances the activation of effector T cells and stabilizes B-T cell interactions, thereby amplifying the local renal inflammatory response. Furthermore, it directly contributes to glomerular complement deposition, promotes renal fibrosis, and exacerbates leukocyte infiltration. These pathological processes collectively result in a further elevation of urinary protein and blood urea nitrogen levels, ultimately worsening renal damage (32). In the advanced stages of DN, B cells contribute directly to the regulation of local renal inflammation through the secretion of pro-inflammatory cytokines such as interleukin-6 (IL-6) and TNF-α. Clinical evidence indicates increased levels of autoreactive B cells in the peripheral blood of T2DN patients. Moreover, the IL-6 secreted by these autoreactive B cells is positively correlated with the degree of renal fibrosis. These findings suggest that the pro-inflammatory activity of B cells may be linked to disease severity in DN (33).

#### 2.1.4 Dendritic cells

DCs, as APCs, play a pivotal role in bridging innate and adaptive immunity through the activation and differentiation of T cells. Based on their distinct phenotypic and functional properties, DCs are broadly categorized into three main subtypes: conventional dendritic cells (cDCs), plasmacytoid dendritic cells (pDCs), and monocyte-derived dendritic cells (moDCs). In the kidneys of healthy individuals, cDCs and pDCs are predominantly located within the renal interstitium, with minimal presence in the glomerular regions. Notably, cDCs represent the predominant subset among renal-resident dendritic cells (34). These DCs can be activated through the recognition of pathogen-associated molecular patterns (PAMPs) or DAMPs. PAMPs are defined as conserved molecular structures derived from microorganisms, including bacterial LPS, viral dsRNA, and fungal cell wall components. As non-self motifs, they are specifically recognized by host pattern recognition receptors (PRRs), triggering critical signaling cascades that launch anti-infective immune defenses. Upon maturation, they upregulate the expression of co-stimulatory molecules such as CD80 and CD86, as well as the chemokine receptor CCR7, and secrete a range of pro-inflammatory cytokines, including TNF-α, interleukin-1β (IL-1β), IL-6, and interleukin-18 (IL-18) (35, 36). These cytokines play a pivotal role in the pathogenesis of DN. In DN, multiple factors—such as hyperglycemia, AGEs, and pro-inflammatory cytokines—act synergistically to activate DCs. Upon activation, DCs exacerbate renal inflammatory responses by secreting pro-inflammatory cytokines and facilitating T cell activation, ultimately resulting in tubulointerstitial injury and fibrosis. Concurrently, activated DCs promote the infiltration of additional immune cells into renal tissues, further amplifying renal damage. Moreover, DCs may directly stimulate renal fibroblasts through the secretion of TGF-β, thereby driving interstitial fibrosis and accelerating the progression of DN (36). Activated dendritic cells interact with macrophages, triggering kidney inflammation by releasing pro-inflammatory cytokines and chemokines. These factors work together to recruit peripheral monocytes to the kidneys and promote their polarization into M1-type macrophages. As key effector cells, M1-type macrophages directly damage renal tubular epithelial cells and mesangial cells, and the cytokines they secrete can also re-activate dendritic cells, forming a positive feedback loop. This loop maintains the activated state of dendritic cells, leading to the continuous recruitment and activation of immune cells, thereby continuously amplifying the local inflammatory response in the kidneys (37). There is a bidirectional regulatory interaction between T cells and DCs. After DCs are activated, they highly express co-stimulatory molecules, which bind to the surface CD28 and CD40L of T cells to provide co-stimulatory signals to activate T cells; Activated T cells can, through surface molecule binding and cytokine secretion, reverse enhance the survival, activation and antigen-presenting ability of DCs. The two form a positive feedback loop to continuously amplify the immune response in the kidneys, ultimately promoting the infiltration of effector T cells into the kidneys and mediating the inflammatory injury and fibrosis of the kidneys (38).

#### 2.1.5 Neutrophils

Neutrophils are a critical first line of defense in the innate immune system. Under hyperglycemic conditions, neutrophils show markedly enhanced activity. As the most abundant inflammatory cell type in peripheral blood, they secrete increased levels of ROS and pro-inflammatory cytokines, including IL-6, interleukin-8 (IL-8), and TNF-α.These mediators promote the recruitment of additional inflammatory cells, thereby exacerbating the local renal inflammatory response in DN (39). In addition, hyperglycemia can trigger neutrophils to release neutrophil extracellular traps (NETs), which primarily consist of chromatin and granular proteins such as myeloperoxidase and elastase. NETs are reticular structures released by activated neutrophils. They are composed of decondensed chromatin, histones, and antimicrobial granule proteins, which enable them to capture and kill pathogens. While crucial for anti-infective immunity, excessive NET formation is closely linked to the pathogenesis of autoimmune diseases, thrombosis, and organ damage. NETs directly damage glomerular endothelial cells and podocytes, leading to their apoptosis and functional impairment. Additionally, they promote renal fibrosis by inducing a phenotypic transition in renal tubular epithelial cells. Together, these mechanisms contribute to the development of DN. Moreover, neutrophil-derived elastase can degrade vascular endothelial (VE)-cadherin, a key intercellular junction protein, disrupt endothelial tight junctions, and consequently increase vascular permeability and albuminuria. These interconnected mechanisms collectively accelerate the onset and progression of DN (40). NETs primarily function to capture and eliminate pathogens. However, in the hyperglycemic environment of diabetic DN, neutrophils become overactivated and release excessive NETs. Components of NETs—such as histones and myeloperoxidase (MPO)—are cytotoxic and can directly damage the cell membranes of glomerular endothelial cells, podocytes, and renal tubular epithelial cells, leading to cell apoptosis or functional impairment. NETs can also induce pyroptosis—a programmed form of cell death—in glomerular endothelial cells. This process causes the release of intracellular contents, which in turn amplifies inflammation and worsens renal tissue injury. Furthermore, NETs secrete pro-inflammatory mediators such as C-X-C motif chemokine ligand 8 (CXCL8) and IL-6. These mediators facilitate the recruitment of more neutrophils and macrophages into renal tissues, thus forming a self-sustaining inflammatory vicious cycle in the kidney. During NET formation, large amounts of ROS are generated. ROS not only directly damage cellular structures but also activate the NLRP3 inflammasome, thereby enhancing the production of pro-inflammatory cytokines and further exacerbating renal injury. Studies have shown that levels of NET-specific markers—such as citrullinated histone H3 (Cit-H3) and the myeloperoxidase-DNA (MPO-DNA) complex—are significantly elevated in the urine and renal tissues of patients with DN. These elevated levels show a positive correlation with both the urinary albumin-to-creatinine ratio (UACR) and the degree of glomerulosclerosis. These findings suggest that NETs may serve as a potential biomarker for disease diagnosis and prognostic evaluation in DN (41, 42).

### 2.2 Non-immune cells

Intrinsic renal non-immune cells, including glomerular cells (such as podocytes, mesangial cells, and endothelial cells) and tubulointerstitial cells (such as renal tubular epithelial cells and renal fibroblasts), are essential constituents of the renal immune microenvironment (**Table 1**). Under hyperglycemic conditions, these cells not only serve as targets of injury but also actively regulate the renal immune microenvironment by releasing pro-inflammatory mediators, upregulating adhesion molecules, and engaging in bidirectional crosstalk with immune cells (43).

**Table 1:** Abnormal functions and regulatory mechanisms of renal non-immune cells in diabetic nephropathy.

Renal non-immune cell types	Abnormal manifestations	Pathways/molecules	Impact on the progression of DN
Renal Tubular Epithelial Cells	Secrete more pro-inflammatory and chemotactic factors Activate programmed cell death Initiate epithelial-mesenchymal transition	NF-κB, MAPK PERK/ATF4/CHAC1 TGF-β/Smad NLRP3 Inflammasome	Exacerbate tubulointerstitial inflammation Amplify local inflammatory responses Contribute to renal interstitial fibrosis
Podocytes	Upregulate chemokine secretion and adhesion molecules Promote the adhesion and infiltration of immune cells Trigger humoral immunity Promote apoptosis	JAK/STAT AGEs TNF-α Nrf2/HO-1 Complement system	Exacerbate glomerular inflammation Disrupt the structure of the glomerulus Induce proteinuria
Mesangial Cells	Increase secretion of pro-inflammatory factors Accumulate excessive extracellular matrix Overexpress complement components Excessive cell proliferation	Complement activation pathway NF-κB TGF-β/Smad PDGF	Recruit immune cells Promote glomerular sclerosis Activate the complement system Exacerbate glomerular inflammation and injury
Glomerular Endothelial Cells	Upregulate adhesion molecule expression Promote inflammatory cell adhesion Increase of pro-inflammatory factors Exacerbate glomerular inflammation Increased permeability Increase the risk of thrombosis	ROS HIF-1α/Notch1 Endothelial-mesenchymal transition-relatedpathways	Promote the adhesion and migration of inflammatory cells Exacerbate glomerular inflammation Lead to elevated glomerular filtration and proteinuria Increase the risk of microthrombosis
Fibroblasts	Activate and differentiate into myofibroblasts Secrete of pro-inflammatory factors Recruit immune cells Upregulate TIMPs and downregulate MMPs Overproduce extracellular matrix	TGF-β/Smad2/3 TIMPs/MMPs PI3K/Akt CTGF	Recruit immune cells and amplify the inflammatory response Inhibit the degradation of extracellular matrix Contribute to renal interstitialfibrosis

In DN, non-immune cells in the kidney promote disease progression through mechanisms such as abnormal activation, secretion of inflammatory factors, cell death, and phenotypic transformation. These cells mediate inflammatory responses, recruitment of immune cells, deposition of extracellular matrix, and fibrosis through key signaling pathways such as NF-κB and TGF-β, ultimately leading to damage and loss of renal function and structure.

#### 2.2.1 Renal tubular epithelial cells

Renal tubular epithelial cells (RTECs) are the primary functional cells responsible for renal reabsorption and secretion, and they play a pivotal role in the pathogenesis of DN. Under hyperglycemic conditions, RTECs contribute to renal immune dysregulation through three major mechanisms: First, secretion of Inflammatory and Chemotactic Factors. Under hyperglycemic conditions, activation of the TLR4 receptor in renal tubular epithelial cells triggers the NF-κB signaling pathway, leading to enhanced expression and release of inflammatory mediators such as MCP-1, IL-6.These factors promote the recruitment and infiltration of inflammatory cells—including macrophages and neutrophils—into the renal tubulointerstitial compartment (44). Second, mediating programmed cell death. Hyperglycemia can activate inflammasomes, thereby upregulating the expression of key pyroptosis-related proteins such as NLRP3 and GSDMD, and enhancing the secretion of pro-inflammatory cytokines including IL-1β and IL-18, which collectively exacerbate local inflammatory responses (45). Third, in DN, hyperglycemia and its associated metabolic derangements serve as the primary initiating factors that induce the EMT in RTECs. These abnormalities activate multiple signaling pathways, including TGF-β and CTGF, thereby promoting the EMT process (46). Persistent activation of the pro-fibrotic mediators TGF-β and CTGF drives renal tubular epithelial cells to progressively shed their epithelial phenotype. This is characterized by loss of cellular polarity, impaired intercellular adhesion, and downregulation of epithelial markers such as E-cadherin. Concurrently, these cells acquire a mesenchymal-like state, marked by upregulation of interstitial markers and enhanced migratory and invasive capacities. Ultimately, these transformed cells can dedifferentiate into myofibroblasts, thereby directly contributing to the development and progression of renal tubulointerstitial fibrosis (46, 47). Renal tubular epithelial cells undergoing EMT not only exhibit compromised structural and functional integrity and diminished regenerative capacity, but also secrete inflammatory mediators that recruit immune cells, thereby establishing a pro-inflammatory microenvironment. This microenvironment, in turn, further exacerbates EMT, creating a vicious cycle that persistently drives the progression of diabetic nephropathy (48).

#### 2.2.2 Podocytes

Podocytes, as a critical component of the glomerular filtration barrier, collaborate with the glomerular basement membrane (GBM) and glomerular endothelial cells to establish an efficient filtration barrier under physiological conditions, thereby preventing the leakage of large-molecular-weight proteins (49). However, in DN, hyperglycemia and other contributing factors disrupt this equilibrium, leading to podocyte injury and subsequent proteinuria, a hallmark pathological feature of disease progression (50). Under hyperglycemic conditions, podocytes also participate in the regulation of the renal immune microenvironment through the following pathways. Firstly, hyperglycemic environment activates the JAK/STAT signaling pathway in podocytes, resulting in significant upregulation of C-X-C motif chemokine ligand 9 (CXCL9) expression. Secreted CXCL9 enters the glomerular microenvironment and binds to its receptor C-X-C chemokine receptor type 3(CXCR3), directly impairing podocyte structural integrity while promoting immune cell infiltration into the glomerulus (51). Moreover, advanced glycation end products enhance MCP-1 expression in podocytes, specifically facilitating macrophage recruitment. Immune cell infiltration disrupts podocyte tight junction structures, aggravating podocyte injury and glomerular filtration dysfunction, thereby perpetuating a vicious cycle of inflammation and cellular damage (52). Secondly, podocytes secrete pro-inflammatory mediators such as IL-6 and TNF-α, which directly activate intraglomerular immune cells and amplify local inflammatory responses (53). Thirdly, podocytes can upregulate the expression of adhesion molecules, including ICAM-1 and VCAM-1, in response to inflammatory stimuli such as IL-1β, TNF-α, IFN-α, and IFN-γ. This enhanced expression promotes the recruitment, adhesion, and infiltration of immune cells—particularly macrophages and lymphocytes—into the glomerular compartment, thereby contributing to the inflammatory pathogenesis observed in glomerular diseases (54).

#### 2.2.3 Mesangial cells

Mesangial cells play a crucial role in maintaining the structural integrity and filtration function of the glomeruli (55). In DN, excessive mesangial cell proliferation and abnormal ECM deposition are hallmark features of glomerulosclerosis. Hyperglycemia activates the NF-κB and TGF-β/Smad signaling pathways in mesangial cells, leading to the upregulation of pro-inflammatory molecules such as IL-1β, IL-6, and MCP-1, both in expression and secretion. These mediators selectively recruit immune cells—including macrophages and T cells—into the glomeruli. Once infiltrated, macrophages release additional inflammatory factors, thereby amplifying local inflammation and establishing a self-sustaining inflammatory loop that progressively disrupts glomerular structure and function (56, 57). Additionally, under the influence of cytokines such as TGF-β and PDGF, mesangial cells produce excessive amounts of collagen types I, III, and IV, along with fibronectin. Under physiological conditions, the synthesis and degradation of glomerular ECM are maintained in a dynamic equilibrium. However, in DN, sustained hyperglycemia disrupts this balance by promoting ECM synthesis while inhibiting its degradation, resulting in net accumulation of ECM within the glomerular mesangium. This progressive deposition leads to mesangial expansion and ultimately contributes to the development of glomerulosclerosis (58). Notably, mesangial cells can express complement components C3 and C5. In DN, these cells exhibit abnormally elevated expression of C3 and C5—changes that are closely linked to the progression of glomerular sclerosis and proteinuria, two hallmark pathological features of DN—leading to activation of the complement system. This activation generates a cascade of complement cleavage products, including C3a and C5a (59). Specifically, C5a binds to C5a receptors on inflammatory cells, triggering their activation and the subsequent release of pro-inflammatory mediators such as ROS and lysosomal enzymes. These mediators directly injure intrinsic glomerular cells—including podocytes and endothelial cells—and damage the GBM, thereby exacerbating glomerular inflammation and injury (60).

#### 2.2.4 Endothelial cells

Glomerular endothelial cells constitute a critical component of the renal filtration barrier. Injury to these cells can result in glomerular hyperfiltration, proteinuria, and microthrombosis, contributing to progressive kidney dysfunction (61). In DN, hyperglycemia initially induces these cells to upregulate adhesion molecules such as ICAM-1 and VCAM-1, promoting monocyte and neutrophil adhesion and transmigration into the glomerular compartment. Concurrently, injured endothelial cells secrete pro-inflammatory cytokines, including IL-6, TNF-α, and CXCL8, thereby amplifying intraglomerular inflammatory responses. Subsequently, hyperglycemia-induced ROS disrupt tight junctions between endothelial cells, increasing glomerular endothelial permeability and contributing to proteinuria. Following injury, endothelial cells upregulate tissue factor expression, while hyperglycemia simultaneously impairs their intrinsic anticoagulant functions, collectively predisposing to microthrombosis. Moreover, a sustained hyperglycemic environment promotes glomerular endothelial cell apoptosis, severely compromising the structural and functional integrity of the glomerular vascular barrier and accelerating DN progression (62, 63).

#### 2.2.5 Fibroblasts

Fibroblasts are the predominant cell type in the renal interstitium, and their activation and proliferation constitute central mechanisms in the development of renal interstitial fibrosis—a hallmark pathological process that drives the progression of DN to ESRD (64). In DN, hyperglycemia and inflammatory mediators act synergistically to activate renal interstitial fibroblasts. Upon stimulation by TGF-β, these activated fibroblasts differentiate into myofibroblasts primarily through the Smad2/3 signaling pathway. Myofibroblasts display a markedly enhanced capacity for ECM production and serve as the principal effector cells in the pathogenesis of renal interstitial fibrosis (65). Subsequently, myofibroblasts secrete inhibitors of matrix metalloproteinases (MMPs), such as tissue inhibitors of metalloproteinases (TIMPs), thereby reducing ECM degradation and exacerbating pathological renal tissue remodeling and interstitial fibrosis. Furthermore, activated renal interstitial fibroblasts release pro-inflammatory cytokines, including IL-6, TNF-α, and MCP-1, which promote immune cell infiltration and amplify inflammatory responses in both glomerular and interstitial compartments. Notably, fibroblasts also produce MMPs and their specific inhibitors, TIMPs. In DN, TIMP expression is upregulated while MMP expression is downregulated. This imbalance between MMPs and TIMPs not only suppresses ECM degradation but also drives excessive ECM accumulation, thereby accelerating the progression of renal interstitial fibrosis (66, 67).

#### 2.2.6 Cytokines and chemokines

Cytokines and chemokines are pivotal effector molecules that regulate cellular functions and inflammatory responses within the immune microenvironment (64). Through the formation of a complex regulatory network, they precisely control the recruitment, activation, and functional behavior of immune cells, while also directly influencing renal intrinsic cells to contribute to the pathological progression of DN. The maintenance of immune microenvironment homeostasis depends on a finely tuned balance between pro-inflammatory and anti-inflammatory mediators. During DN progression, the expression levels of multiple pro-inflammatory cytokines and chemokines are markedly elevated (68).

##### 2.2.6.1 Pro-inflammatory cytokines

Pro-inflammatory cytokines are markedly upregulated in DN, where they play pivotal roles in initiating and amplifying inflammatory responses, recruiting immune cells to sites of injury, and driving the progression of renal fibrosis (64). Key mediators include the following: TNF-α, primarily secreted by macrophages and T cells, activates the NF-κB signaling pathway, thereby promoting the expression of chemokines such as MCP-1 and adhesion molecules including ICAM-1 in renal intrinsic cells. It also exerts direct cytotoxic effects on podocytes and renal tubular epithelial cells, contributing to the progression of glomerulosclerosis and tubulointerstitial fibrosis. Clinical evidence has demonstrated a positive correlation between serum TNF-α levels and the rate of eGFR decline in patients with DN, and targeted inhibition of TNF-α has been shown to attenuate renal injury in animal models (69, 70). IL-1β is primarily secreted by activated macrophages and dendritic cells through the NLRP3 inflammasome pathway and exerts potent pro-inflammatory effects. It activates glomerular mesangial cells and renal tubular epithelial cells, promoting the expression of pro-inflammatory mediators such as IL-6 and MCP-1. Furthermore, IL-1β induces fibroblast activation, contributing to renal interstitial fibrosis. It also stimulates T and B lymphocytes, thereby enhancing adaptive immune responses (33, 71). IL-6, secreted by macrophages, T cells, and renal intrinsic cells, activates the JAK/STAT3 signaling pathway to promote Th17 cell differentiation, suppress Treg cell function, and stimulate glomerular mesangial cells to synthesize ECM, thereby exacerbating renal inflammation and fibrosis (71, 72).

##### 2.2.6.2 Chemokines

Chemokines are pivotal molecules that mediate the directional migration of immune cells. In DN, their upregulated expression constitutes a central mechanism driving the selective infiltration of immune cells into the kidneys (73). ① Monocyte chemoattractant protein-1 (MCP-1/CCL2), the most extensively studied chemokine, is primarily produced by intrinsic renal cells—such as podocytes, mesangial cells, and renal tubular epithelial cells—as well as by macrophages, and plays a pivotal role in the recruitment of monocytes and macrophages. Elevated expression of MCP-1 has been consistently documented in patients with DN, with levels showing a positive correlation with the degree of renal macrophage infiltration and tubular injury, thus serving as a reliable biomarker of tubular damage severity. Under hyperglycemic conditions, signaling pathways including TLR4/NF-κB and TAK1/MAPK are activated, leading to increased CCL2 secretion by glomerular podocytes, mesangial cells, and renal tubular epithelial cells. Furthermore, AGEs and aldosterone can upregulate CCL2 expression via the NF-κB pathway, thereby exacerbating inflammatory responses, promoting ECM accumulation, and accelerating the progression of renal fibrosis (74). ② CXCL8 (IL-8) is predominantly secreted by renal tubular epithelial cells and endothelial cells. It binds to CXCR1 and CXCR2 receptors on neutrophils, thereby promoting their recruitment into the kidneys and enhancing local inflammatory responses. Under specific conditions, CXCL8 also modulates endothelial cell adhesion and mediates the chemotaxis and activation of additional leukocyte subsets, such as monocytes stimulated by IL-13 or IL-4, certain CD8^+^T lymphocytes, and mast cells (75).

##### 2.2.6.3 Anti-inflammatory cytokines

Anti-inflammatory cytokines primarily consist of IL-10 and IL-4, which are secreted by Treg and Th2 cells, respectively (76). Anti-inflammatory cytokines, mainly interleukin-10 and interleukin-4, play a crucial role in regulating renal inflammation. Interleukin-10 reduces the expression of pro-inflammatory factors and inhibits the polarization of M1-type macrophages by suppressing the NF-κB signaling pathway, thereby exerting anti-inflammatory and tissue repair effects. Interleukin-4, on the other hand, promotes the polarization of macrophages towards the repair-promoting M2-type phenotype. In DN, the expression levels of these two cytokines are significantly reduced (33, 77).

##### 2.2.6.4 Other factors

Additional factors play significant roles in the pathogenesis and progression of DN. For instance, mesenchymal stem cells (MSCs) have been shown to secrete anti-inflammatory mediators such as lipoxin A4 (LXA4), which suppress pro-inflammatory cytokines and enhance renal homeostasis, contributing to the amelioration of DN (78).

### 2.3 Extracellular matrix

The ECM functions as the structural framework of renal tissue and is primarily composed of key components including collagens (types I, III, and IV), fibronectin, and laminin. The maintenance of ECM homeostasis is essential for preserving both the structural integrity and physiological function of the kidney (79). In DN, hyperglycemia and dysregulation of the immune microenvironment synergistically promote ECM accumulation by enhancing ECM synthesis and impairing its degradation, resulting in aberrant ECM remodeling—a central mechanism underlying renal fibrosis. Hyperglycemia activates key signaling pathways, including TGF-β/Smad and PI3K/Akt, in intrinsic renal cells such as mesangial cells and fibroblasts, thereby stimulating the production of ECM components like collagen and fibronectin (80). Meanwhile, pro-fibrotic factors such as TGF-β and CTGF, secreted by immune cells including M2 macrophages and Th17 cells, further promote ECM synthesis (81).

Additionally, ECM degradation primarily depends on MMPs, whose activity is regulated by tissue inhibitors of TIMPs. In DN, hyperglycemia and inflammatory cytokines upregulate TIMP expression and suppress MMP activity, resulting in diminished ECM turnover, impaired degradation, and subsequent accumulation of ECM components (82). Excessively deposited ECM alters the biomechanical properties of the local microenvironment, which promotes macrophage polarization toward the M2 phenotype and facilitates Th17 cell differentiation—both of which contribute to the progression of fibrosis. Furthermore, ECM degradation products, such as collagen fragments, function as DAMPs that activate DCs and macrophages, thereby inducing the release of pro-inflammatory cytokines and amplifying chronic inflammatory responses (83, 84).

## 3 Mechanisms of macrophages in the progression of diabetic nephropathy

Macrophages play a pivotal role in the progression of DN. These cells demonstrate remarkable plasticity, dynamically adapting their functional phenotypes in response to alterations in the local microenvironment. Depending on specific environmental signals, macrophages can be polarized into two distinct phenotypes, classically activated M1 macrophages and alternatively activated M2 macrophages (85). M1 macrophages primarily secrete pro-inflammatory cytokines and chemokines, thereby recruiting immune cells and amplifying inflammatory responses. In contrast, M2 macrophages produce anti-inflammatory cytokines that help regulate and suppress excessive immune activation. In the context of diabetic renal injury, M1 macrophages are the predominant subtype, contributing to sustained inflammation and tissue damage (86).

### 3.1 Macrophage polarization

The polarization and functional activities of macrophages are critically involved in the progression of DN. M1 macrophages contribute to disease advancement through multiple molecular and cellular pathways. Under conditions of hyperglycemia and oxidative stress, intrinsic renal cells produce elevated levels of MCP-1, which promotes the recruitment of circulating monocytes into the kidney. These infiltrating monocytes subsequently differentiate into M1 macrophages, amplifying local inflammatory responses and exacerbating renal injury (87). In diabetic kidneys, the expression of adhesion molecules such as ICAM-1 and VCAM-1 is markedly upregulated. These molecules enhance monocyte adhesion to renal endothelial cells, thereby promoting transendothelial migration into renal tissues, where monocytes differentiate into M1 macrophages and contribute to disease progression. Moreover, under conditions of hyperglycemia and oxidative stress, M1 macrophages secrete substantial levels of pro-inflammatory cytokines, triggering local inflammatory responses and recruiting additional immune cells to the kidney. This creates a self-perpetuating cycle that exacerbates renal injury (88). Moreover, stress-induced products such as ROS generated under conditions of hyperglycemia and oxidative stress not only exert direct cytotoxic effects on renal cells but also act synergistically with pro-inflammatory mediators to amplify inflammatory responses (89). Inflammatory mediators released by M1 macrophages can directly damage intrinsic renal cells, leading to cellular injury or even necrosis, and thereby disrupting normal renal architecture. The cytokines and chemokines secreted by these cells promote the synthesis of excessive ECM proteins—such as collagen and fibronectin—in renal tissues. Accumulation of these ECM components in the kidney results in glomerulosclerosis and renal interstitial fibrosis, which are hallmark pathological features of advanced DN (63). M1 macrophages also contribute to the progression of DN by participating in immune dysregulation. They possess antigen-presenting capacity, which enables them to present renal tissue-derived antigens to adaptive immune cells. This process activates immune responses, induces excessive activation and massive accumulation of local immune cells, and ultimately augments immune-mediated renal injury. Furthermore, cytokines secreted by M1 macrophages can modulate the functions of other immune cells—for example, promoting the polarization of monocytes into M1 macrophages and inhibiting the activity of Treg cells. Collectively, these effects disrupt local immune homeostasis and drive the progression of DN (14, 90).

The role of M2 macrophages in DN is complex and dualistic. On one hand, M2 macrophages secrete profibrotic factors such as TGF-β and FGF. These factors activate myofibroblasts, promote their proliferation, and ultimately lead to excessive collagen deposition in the ECM and subsequent renal fibrosis. Moreover, M2 macrophages can undergo transdifferentiation into mesenchymal-like cells that acquire mesenchymal characteristics, thereby contributing to ECM synthesis and secretion and exacerbating fibrotic progression (91). Excessive M2 macrophage polarization promotes mesangial proliferation and basement membrane thickening, alters the renal microenvironment, and disrupts the physiological functions of intrinsic renal cells including podocytes and tubular epithelial cells. These changes impair the glomerular filtration barrier, ultimately leading to renal interstitial fibrosis and glomerulosclerosis, which accelerates the progression of renal dysfunction (17). Type M2 macrophages exert anti-inflammatory and tissue repair effects locally in the kidneys by producing anti-inflammatory cytokines such as IL-10 (92). They not only regulate inflammatory responses and alleviate acute inflammatory damage to the glomeruli and renal tubules, but also directly inhibit the activation of M1-type macrophages and the release of pro-inflammatory factors like TNF-α and IL-1β, thereby suppressing the amplification of chronic inflammation. Moreover, M2-type macrophages can reduce the excessive infiltration of immune cells in the renal tissue, overall alleviating the inflammatory damage to the kidneys and promoting repair (91). By secreting factors like vascular endothelial growth factor (VEGF) and FGF, M2 macrophages promote renal tubular repair and glomerular microvascular reconstruction, thereby restoring renal structure and function to alleviate the symptoms of DN (93). Furthermore, M2 macrophages utilize their strong phagocytic capacity to clear cellular debris and immune complexes from the kidneys. This clearance limits the release of pro-inflammatory mediators and helps maintain local tissue microenvironment stability, thereby facilitating the recovery of damaged renal tissue (94). Functioning as the central mediators of inflammation resolution and tissue repair, M2 macrophages are classified into four distinct subtypes (M2a, M2b, M2c, and M2d). Their specialized roles are integrated through collaboration, thereby forming a highly organized repair network (95). M2a is a classic type of reparative anti-inflammatory and tissue regeneration. Its main secreted factors are IL-10, TGF-β and chemokines. The core function is to remove cell debris, promote cell growth and tissue regeneration, and inhibit excessive inflammation to maintain homeostasis. As an immunomodulatory macrophage subtype, M2b simultaneously secretes both pro-inflammatory factors and the anti-inflammatory cytokine IL-10. This enables it to moderately activate immune responses while balancing inflammation to prevent excessive tissue damage—a process most commonly observed during the mid-phase of inflammation. The M2c macrophage, a phagocytic and clearance subtype, not only clears apoptotic substances to initiate repair but also secretes key mediators like IL-10 and TGF-β in later stages to drive tissue remodeling and inhibit immune overactivation, thereby restoring homeostasis. Angiogenic M2d macrophages promote new blood vessel formation by secreting VEGF and IL-10, thereby improving local perfusion. By acting synergistically with other M2 subtypes, they enhance the overall tissue repair network, which contributes to reducing the risk of kidney fibrosis (95, 96).

#### 3.1.1 Interaction between macrophages and renal cells

There is a highly intricate crosstalk between macrophages and renal intrinsic cells. Under pathological stimuli such as hyperglycemia, renal tubular epithelial cells secrete a range of pro-inflammatory cytokines, including IL-1, IL-6, and TNF-α. These cytokines exert effects on both renal tubular epithelial cells and infiltrating macrophages, triggering local inflammatory responses that result in cellular damage and functional impairment, thereby promoting the progression of DN (33). When renal tubular epithelial cells are injured or in a pro-inflammatory state, they upregulate the secretion of specific chemokines—most notably MCP-1—which facilitate the recruitment and accumulation of macrophages in the kidney. As macrophage infiltration intensifies, these immune cells release additional pro-inflammatory mediators, establishing a self-amplifying feedback loop that exacerbates inflammation and tubulointerstitial injury, ultimately accelerating the advancement of DN (33, 97).

During the progression of DN, glomerular cells are profoundly influenced by macrophage activity. Macrophages release pro-inflammatory cytokines, including IL-1, IL-6, and TNF-α, which stimulate glomerular mesangial cells to overproduce ECM, thereby accelerating glomerulosclerosis and worsening glomerular injury (16). Upon glomerular damage or inflammation, MCP-1 is upregulated, promoting the recruitment of substantial numbers of macrophages into the glomerular compartment. Once infiltrated, these macrophages secrete additional pro-inflammatory mediators that further impair glomerular cells, establishing a self-perpetuating cycle of injury. Under hyperglycemic conditions, hypoxia-inducible factor-1α (HIF-1α) and Notch1 signaling pathways in glomerular endothelial cells are activated. This activation directly enhances the expression of chemotactic molecules in endothelial cells, leading to the selective recruitment of pro-inflammatory M1 macrophages to the kidneys, which exacerbates endothelial dysfunction and disrupts the glomerular microenvironment (62). In this hyperglycemic milieu, podocytes also secrete MCP-1, facilitating macrophage migration into renal tissue. Following infiltration, macrophages produce TNF-α, which induces podocyte apoptosis. Moreover, activation of T-cell immunoglobulin and mucin domain-containing molecule 3 (Tim-3) triggers macrophages to upregulate NF-κB and TNF-α production, amplifying local inflammatory responses and hastening podocyte injury (16). Metabolically, AGEs promote macrophage polarization toward the pro-inflammatory M1 phenotype. These polarized M1 macrophages sustainably release high levels of pro-inflammatory cytokines, altering glomerular function and thereby driving the progression of DN (86).

### 3.2 Mechanisms of T cells in the progression of diabetic nephropathy

T cells contribute to the pathogenesis of DN through multiple mechanisms, including promoting inflammatory responses, exerting direct cytotoxic effects, and mediating immunoregulatory functions. Together, these actions exacerbate renal injury in DN (98).

#### 3.2.1 Pro-inflammatory effects

T cells secrete a variety of pro-inflammatory cytokines. Specifically, IFN-γ, interleukin-2 (IL-2), and TNF-α produced by Th1 cells can activate macrophages and other immune cells, thereby exacerbating kidney injury in DN (77). In DN, an increased proportion of Th17 cells is closely associated with the severity of renal damage. IL-17, a key pro-inflammatory cytokine secreted by Th17 cells, stimulates various cell types to produce chemokines and inflammatory mediators, promoting inflammatory cell infiltration and sustaining inflammation. During the pathogenesis of diabetic nephropathy, upregulated IL-17 expression induces renal glomerular mesangial cells and other resident renal cells to secrete higher levels of inflammatory chemokines. These chemokines recruit immune cells into the kidney, thereby amplifying the local inflammatory response. Moreover, Th17 cells interact with other renal resident cells to further propagate inflammation. For example, IL-17 signaling in renal tubular epithelial cells triggers aberrant expression of inflammation-related genes, leading to tubulointerstitial inflammation and contributing to the progression of kidney injury (23).

A substantial population of CD8^+^T cells secrete a range of pro-inflammatory cytokines, notably IFN-γ and TNF-α, which activate macrophages and other immune effector cells. This activation promotes the persistent recruitment of inflammatory cells and the excessive release of inflammatory mediators, thereby exacerbating renal inflammation and contributing to progressive tissue injury (99). In parallel, CD8^+^T cells release cytotoxic molecules, including perforin and granzyme B. Granzyme B is internalized by target cells and induces apoptosis, directly compromising renal cellular integrity, triggering cell death, and leading to structural damage that ultimately undermines normal kidney function (77).

#### 3.2.2 Immune regulatory effects and immune tolerance imbalance

Alterations in the numbers of T cell subsets also play a pivotal role in the progression of diabetic nephropathy. Tregs are critical for regulating immune responses and maintaining immune tolerance; impaired Treg function leads to excessive activation of Th17 cells, thereby increasing the risk of glomerular sclerosis. Meanwhile, hyperglycemia-induced oxidative stress can impair Tregs, reducing the secretion of TGF-β and IL-10 and consequently attenuating their anti-inflammatory regulatory effects (25).

The number of Th1 cells is significantly increased in DN. These cells secrete IFN-γ and TNF-α, which exacerbate inflammation and promote pro-inflammatory processes, ultimately inducing renal tubular cell apoptosis and podocyte loss (100). These changes are closely associated with the progressive decline in renal function and further amplify the systemic inflammatory state. Meanwhile, a reduction in Th2 cell numbers impairs the body’s intrinsic anti-inflammatory capacity, thereby aggravating renal inflammation and injury (25, 90). The relatively low proportion of Th2 cells contributes to a Th1/Th2 imbalance, which further intensifies inflammatory responses.

In DN, an elevated proportion of Th17 cells is strongly correlated with the severity of renal injury. IL-17 secreted by Th17 cells can stimulate renal tubular epithelial cells to produce chemokines, which recruit substantial numbers of neutrophils and thereby exacerbate renal tissue damage (101). Moreover, Th17 cells can activate renal fibroblasts to synthesize collagen, directly contributing to the progression of renal interstitial fibrosis (102).

#### 3.2.3 Interaction between T cells and renal cells

Cytokines secreted by T cells, such as IL-2, IFN-γ, and TNF-α, can activate macrophages and trigger inflammatory responses. This promotes inflammatory cell infiltration in the glomeruli and renal tubular interstitium, thereby exacerbating renal tissue damage (25, 77). In addition, T cells release cytotoxic molecules that directly target renal cells, leading to cellular injury or death. Notably, these molecules interact with podocytes and mediate the release of perforin and granzymes, which disrupt podocyte cell membranes and intracellular organelles. Consequently, podocyte injury and detachment occur, compromising the integrity of the glomerular filtration barrier and ultimately contributing to the development of proteinuria (77).In addition to direct injury, T cells can indirectly impair renal podocytes through multiple pathways, including cytokine secretion, promotion of immune complex deposition, and induction of metabolic disturbances (103). Immune mediators released by T cells, such as IL-17, stimulate the activation of glomerular mesangial cells and vascular endothelial cells, leading to the production of inflammatory mediators and extracellular matrix components. These factors disrupt normal podocyte growth and metabolic functions, resulting in podocyte injury. Furthermore, T cell-mediated immune responses enhance the deposition of immune complexes in the glomerular basement membrane, triggering complement system activation and inflammatory reactions that exacerbate podocyte damage (21). Additionally, abnormal T cell activation may trigger localized exacerbation of oxidative stress and disturbances in lipid metabolism, which can impair the normal physiological functions of podocytes and ultimately lead to cellular damage.

#### 3.2.4 Interaction between T cells and other immune cells

Studies have demonstrated a significant increase in renal T cell infiltration in animal models of diabetic nephropathy. Under hyperglycemic conditions, T cells secrete a variety of chemokines and cytokines that not only initiate inflammatory responses but also activate macrophages and endothelial cells, leading to impaired renal function through multiple pathways (77). T cells are closely associated with macrophages, cytokines secreted by T cells can activate renal resident macrophages, prompting glomerular mesangial cells to produce colony-stimulating factor-1 (CSF-1) and MCP-1. These factors in turn amplify macrophage-driven release of a cascade of inflammatory mediators, such as NO, ROS, IL-1, TNF-α, complement components, and MMPs, ultimately exacerbating renal injury (104). Within T cell subsets, Th17 cells engage in intricate and bidirectional interactions with neutrophils, and these two cell types act synergistically to promote renal interstitial fibrosis, ultimately leading to significant impairment of renal function (105). Among the mediators involved, IL-17A secreted by Th17 cells plays a pivotal role. IL-17A can bind to specific receptors on the surface of renal interstitial cells, initiating chemotactic signaling pathways. Additionally, IL-17A induces the expression of various chemokines, including CXCL1, CXCL2, and CXCL8, which exert potent chemotactic effects on neutrophils and recruit large numbers of neutrophils from the systemic circulation to accumulate in the renal interstitium (106). Notably, IL-17A also acts directly on renal epithelial and endothelial cells, inducing them to secrete additional pro-inflammatory mediators and chemokines. This further enhances the chemotaxis and activation of neutrophils. Once activated, these neutrophils release toxic substances such as elastase and myeloperoxidase, directly damaging renal tissue and exacerbating both the inflammatory response and subsequent tissue injury (107, 108).

## 4 Renal immune micro-environment

**Figure 2:**
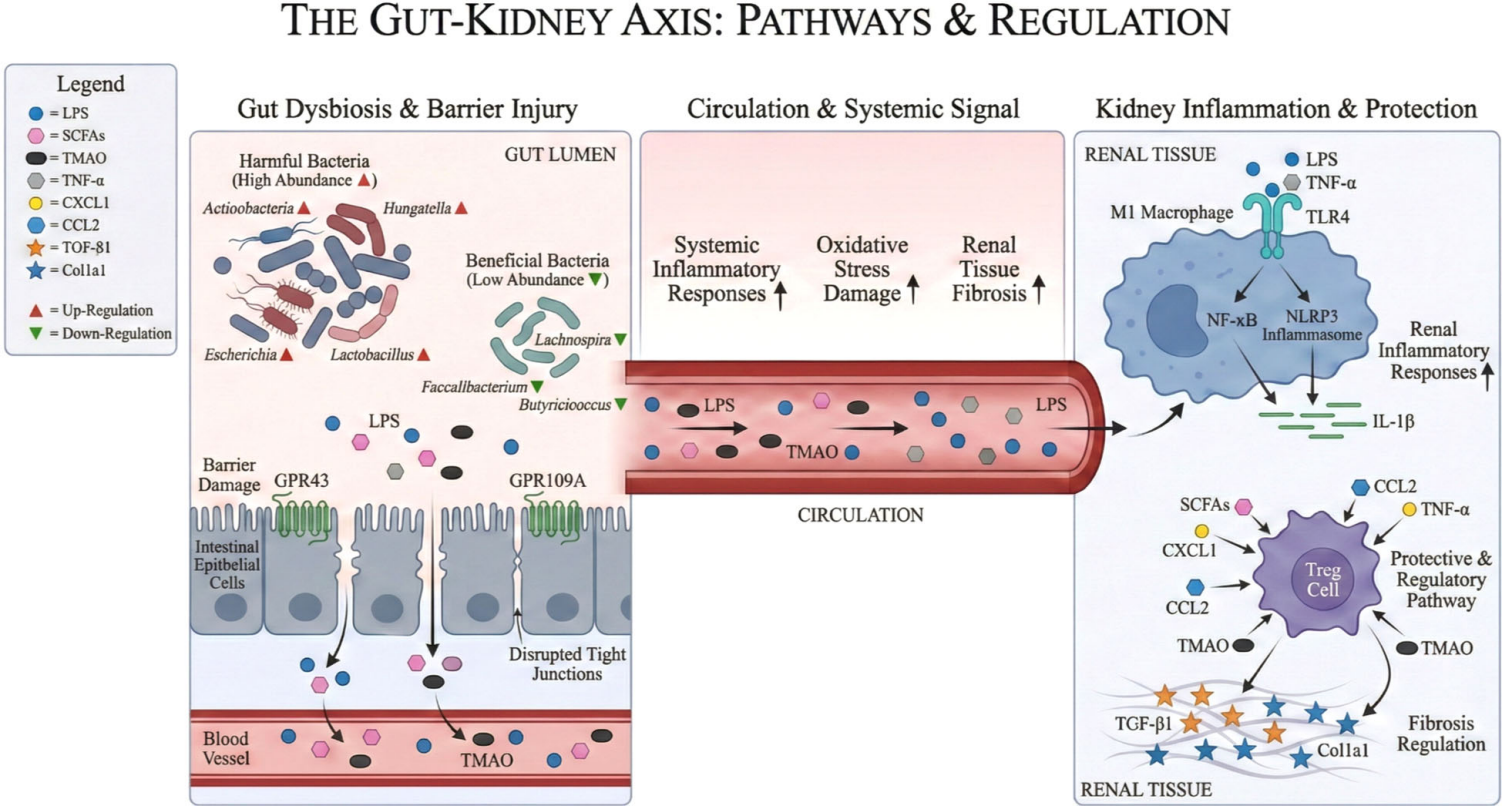
The molecular regulatory network of intestinal flora imbalance mediating renal inflammation and fibrosis. Intestinal flora dysbiosis compromises the gut barrier, allowing bacterial metabolites to enter the circulation. These metabolites activate macrophage TLR4 receptors, triggering the NF-κB and NLRP3 pathways to release pro-inflammatory mediators (TNF-α, IL-1β, CXCL1, CCL2). This promotes renal inflammation, oxidative stress, and the expression of pro-fibrotic factors (Col1α1, TGF-β1), ultimately driving renal fibrosis. Although metabolites like SCFAs can exert anti-inflammatory effects via GPR43/GPR109A, these are insufficient to counteract the dominant pathologic processes in dysbiosis.

Under physiological conditions, the intestinal microbiota maintains a relatively stable dynamic equilibrium. However, during disease states or other perturbations, this equilibrium can be disrupted, leading to dysbiosis (109). In recent years, research has demonstrated that the gut microbiota and its metabolic byproducts play a significant role in the pathogenesis and progression of diabetic nephropathy (**Figure 2**). Patients with diabetes frequently exhibit intestinal dysbiosis, characterized by alterations in both microbial composition and metabolite profiles. A key feature of this dysbiosis is reduced overall microbial diversity, accompanied by marked shifts in the abundance of specific bacterial genera. Notably, the abundance of Gram-negative bacteria such as *Actinobacteria, Hungatella, Escherichia*, and *Lactobacillus* is increased. Among these, the enrichment of *Hungatella* and *Escherichia* is particularly prominent in individuals with diabetic nephropathy. Conversely, beneficial SCFA-producing genera—such as *Butyricicoccus*, *Faecalibacterium*, and *Lachnospira*—are significantly depleted, leading to impaired intestinal anti-inflammatory capacity and disrupted metabolic regulation (110–112). This microbial community imbalance can compromise intestinal barrier integrity, enhance intestinal permeability, and facilitate the translocation of microbial metabolites into the systemic circulation. Once in circulation, these metabolites trigger both systemic and renal-localized immune responses, leading to chronic low-grade inflammation and consequent remodeling of the renal immune microenvironment (113, 114). Under conditions of intestinal microbiota dysbiosis, Lipopolysaccharide (LPS) derived from Gram-negative bacteria translocates into the systemic circulation via a compromised intestinal barrier. Once in circulation, LPS binds to toll-like receptor 4 (TLR4) on macrophages and dendritic cells, triggering activation of the NF-κB pathway and the NLRP3 inflammasome. This sequential signaling cascade enhances the production and release of pro-inflammatory cytokines, including TNF-α and IL-1β, thereby amplifying renal inflammatory responses (114). SCFAs, produced by gut microbiota through the fermentation of dietary fiber, exert renoprotective effects. Specifically, SCFAs mediate the activation of G protein-coupled receptors (GPRs) 43 and 109A. This leads to the downregulation of pro-inflammatory cytokines, chemokines, and pro-fibrotic proteins in diabetic kidneys. Concurrently, SCFAs promote regulatory T cell (Treg) differentiation and help maintain immune homeostasis (115). SCFAs can alleviate renal fibrosis by inhibiting the activation of mesangial cells and fibroblasts. Experimental evidence has confirmed that a high-fiber diet significantly reduces proteinuria, glomerular hypertrophy, podocyte injury, and renal interstitial fibrosis in diabetic mice, and it simultaneously modulates the composition of the gut microbiota (116). TMAO, another metabolite of the gut microbiota, can directly impair renal function through multiple mechanisms. Firstly, it promotes systemic inflammation, exacerbates oxidative stress-induced injury, and induces tissue fibrosis, thereby directly compromising renal structure and function. Secondly, TMAO activates key inflammatory pathways, triggering the release of pro-inflammatory cytokines and directly inducing vascular endothelial dysfunction. Both systemic inflammation and endothelial dysfunction play central roles in the pathogenesis of DN, further supporting the role of TMAO as a key mediator linking gut microbiota dysbiosis to DN progression (117).

In summary, the crosstalk between the gut microbiota and the renal immune microenvironment plays a critical role in the pathogenesis of DN. A deeper understanding of this bidirectional interaction may pave the way for novel therapeutic strategies targeting the gut microbiota to prevent and treat DN. Accumulating evidence has shown that modulation of the gut microbiota—through interventions such as fecal microbiota transplantation (FMT), probiotic supplementation, and dietary prebiotic intake—can effectively alleviate the progression of DN (118–120). However, no large-scale clinical trials have been conducted to provide robust evidence supporting these therapeutic strategies. Therefore, extensive clinical research remains necessary to validate their efficacy, and further in-depth studies are needed to clarify the underlying mechanisms.

## 5 Immune microenvironment markers and diabetic nephropathy

The traditional diagnosis of diabetic nephropathy relies on the assessment of UACR and the rate of decline in glomerular filtration rate (GFR). However, these indicators only show significant changes after kidney damage has occurred, lacking sensitivity and specificity for early diagnosis (121). Therefore, the identification of earlier and more precise biomarkers is crucial to enabling early intervention in diabetic nephropathy and slowing disease progression. Dynamic changes in the renal immune microenvironment provide a promising avenue for discovering novel biomarkers at the early stages of the disease. Biomarkers derived from this microenvironment can not only facilitate early diagnosis but also support prognosis assessment and therapeutic monitoring.

Single-cell RNA sequencing (scRNA-seq) has emerged as a powerful tool for analyzing gene expression profiles across diverse renal cell types under DN conditions. This advancement enables more precise identification of cell-type-specific marker genes (122, 123). The expression levels of FSTL1, CX3CR1, and AGR2 are significantly upregulated in both patients with DN and in DN mouse models. Receiver operating characteristic (ROC) curve analysis has indicated that these genes possess strong diagnostic potential, with area under the curve (AUC) values of 0.911 for FSTL1, 0.935 for CX3CR1, and 0.922 for AGR2, respectively (124). With regard to the functions and diagnostic potential of these genes, the details are as follows: FSTL1 is predominantly expressed in podocytes and mesangial cells. Its expression is significantly upregulated in DN and positively correlated with serum creatinine levels while negatively correlated with GFR. This gene demonstrates strong diagnostic performance. CX3CR1 is highly expressed in leukocytes and plays a critical role in DN pathogenesis by promoting ECM synthesis. Inhibition of CX3CR1 in mouse models of DN reduces ECM deposition, attenuates renal macrophage infiltration, and alleviates renal fibrosis. Given its close association with the progression of renal injury, CX3CR1 may serve as a valuable biomarker for assessing disease prognosis (124). Studies have shown that serum IL-17A levels in patients with diabetic nephropathy DN are positively correlated with UACR and eGFR. Decreased serum IL-17A levels and increased thyroid peroxidase antibody (TPOAb) levels may serve as potential serological biomarkers for both the diagnosis of DN and the assessment of disease severity. The measurement of these biomarkers in patient serum, when combined with other clinical parameters, facilitates more accurate identification of DN and evaluation of renal injury severity, thereby supporting early diagnosis and effective disease monitoring. Given that IL-17A and TPOAb levels are independent predictors of prognosis in DN patients, their assessment contributes to improved risk stratification and clinical decision-making (125).Using untargeted metabolomics analysis via ultra-performance liquid chromatography-tandem mass spectrometry (UPLC-MS/MS), researchers compared patients with type 2 diabetes with and without nephropathy. This approach identified four core potential biomarkers associated with the pantothenic acid and coenzyme A (CoA) biosynthesis pathway. Among these biomarkers, pantothenic acid (vitamin B5), a key pathway intermediate, demonstrated strong diagnostic accuracy for early DN detection. It achieved an area under the AUC of 0.88, with sensitivity and specificity both exceeding 80%, representing the best individual performance among the identified biomarkers. Furthermore, a combined diagnostic model incorporating three biomarkers—pantothenic acid, dihydrouracil, and ureidopropionic acid—improved diagnostic efficacy, yielding an AUC of 0.94, which significantly surpasses conventional renal function markers such as serum creatinine and urine albumin-to-creatinine ratio in distinguishing DN at an early stage (126). In the field of metabolic diseases such as diabetic nephropathy, functional metabolomics has effectively identified multiple key metabolites with clinical translational potential and elucidated their underlying pathological mechanisms, thereby providing robust support for disease research. Alterations in plasma and urine levels of L-carnitine, a precursor of gut microbiota-derived metabolites, correlate with early renal filtration dysfunction and may serve as a potential biomarker for early-stage kidney damage. Phenyl sulfate, another critical metabolite implicated in diabetic nephropathy, is significantly elevated in both *db/db* diabetic mice and patients with the disease. This metabolite exacerbates renal injury through activation of the NF-κB inflammatory pathway, offering direct experimental evidence for the “gut microbiota–metabolite–renal inflammation” axis in diabetic nephropathy and presenting novel candidate targets for early diagnosis and therapeutic intervention (127).

The identification of these biomarkers offers a promising foundation for the early diagnosis, prognosis evaluation, and monitoring of therapeutic efficacy in DN. Nevertheless, their translation into clinical practice necessitates further validation through large-scale, multicenter clinical trials to confirm their effectiveness, reliability, and practical applicability in real-world settings.

## 6 Therapeutic strategies targeting the immune microenvironment

Given the pivotal role of the immune microenvironment in the progression of DN, therapeutic strategies targeting this microenvironment have emerged as a promising avenue for DN treatment. These interventions aim to mitigate renal injury and retard disease progression through modulation of immune cell activity, suppression of inflammatory responses, and potential remodeling of the gut microbiota.

### 6.1 Regulation of macrophage function

Inhibition of Macrophage Recruitment. Given the central role of macrophages in the inflammatory and fibrotic processes of DN, suppressing macrophage recruitment and activation constitutes a pivotal therapeutic approach. Specifically, targeting the recruitment and activation of macrophages can effectively attenuate renal inflammation and fibrosis, primarily by blocking chemokines and their receptors to inhibit monocyte infiltration into the kidneys (128). Evidence indicates that CCR2 is expressed on macrophages, dendritic cells, and T cells. Following renal injury, MCP-1 is upregulated and secreted. The binding of MCP-1 to CCR2 triggers the recruitment of CCR2-expressing monocytes/macrophages, dendritic cells, and fibroblasts to the site of injury, thereby exacerbating renal inflammation and fibrosis. Pharmacological inhibition of the MCP-1/CCR2 axis using CCR2 antagonists has been shown to suppress monocyte migration and infiltration into the kidneys. In preclinical animal models, CCR2 antagonists have significantly reduced renal macrophage accumulation, alleviated inflammatory responses, and ameliorate renal fibrosis (129).

Regulation of Macrophage Polarization. Modulating the polarization status of macrophages and promoting their transition to the anti-inflammatory, reparative M2 phenotype represents a promising therapeutic strategy. This shift can be achieved through genetic editing to regulate macrophage polarization or by engineering macrophages to stably overexpress neutrophil gelatinase-associated lipocalin (NGAL), which helps maintain the M2 phenotype. These genetic modifications enhance the secretion of the anti-inflammatory cytokine IL-10 and reduce TGF-β1 expression in renal tissue. Consequently, they attenuate renal infiltration of M1 macrophages, alleviate podocyte loss and fibrosis, and ultimately delay DN progression (130). Chemical agents or natural plant-derived compounds can modulate macrophage polarization by targeting specific signaling pathways and regulating the secretion of inflammatory cytokines. For instance, active vitamin analogs suppress macrophage polarization toward the pro-inflammatory M1 phenotype and promote a shift to the anti-inflammatory M2 phenotype. This phenotypic switch ameliorates high glucose-induced podocyte injury, restores glomerular filtration barrier function, and reduces proteinuria (16).

Inhibition of Macrophage Activation. Accumulating evidence has demonstrated that aberrant activation of Src homology region 2 domain-containing phosphatase 2 (SHP2) in macrophages plays a critical role in the pathogenesis of DN. SHP2 regulates the mitogen-activated protein kinase (MAPK) and NF-κB signaling pathways, thereby promoting the production and secretion of pro-inflammatory cytokines, which exacerbate renal inflammation and fibrosis. Targeted inhibition of SHP2 in macrophages significantly attenuates DN progression, highlighting its potential as a novel therapeutic strategy for macrophage-directed intervention in DN (131).

### 6.2 Regulation of T cell responses

Modulating the balance and functional competence of T cells represents a promising therapeutic strategy for DN. Treg cells exert immunosuppressive effects by secreting anti-inflammatory cytokines such as IL-10 and TGF-β, thereby inhibiting the activation and proliferation of effector T cells and mitigating renal inflammatory injury (132). Accumulating evidence indicates that the expansion of regulatory T cells (Tregs) can effectively mitigate renal injury through multiple mechanisms. These cells suppress tissue fibrosis and preserve renal architectural integrity. At a molecular level, they downregulate biomarkers associated with injury, inflammation, and fibrosis. Furthermore, Treg expansion attenuates the damage-triggered inflammatory response and reduces the production of damage-associated molecular mediators (133). Conversely, inhibition of Th17 cell differentiation also confers therapeutic benefits in DN. IL-17A, secreted by Th17 cells, acts synergistically with high glucose to promote the expression of inflammatory cytokines and chemokines, thereby exacerbating renal cell injury. Specifically, IL-17A enhances the upregulation of IL-6, TNF-α, and CCL2 in mesangial and renal tubular epithelial cells, leading to increased recruitment of local macrophages and amplification of the inflammatory cascade. Moreover, IL-17A promotes mesangial expansion and renal fibrosis, induces podocyte inflammation and apoptosis—processes closely linked to albuminuria. Therefore, targeted inhibition of IL-17A and suppression of Th17 cell differentiation and activity may effectively alleviate key pathological features of DN (102).

### 6.3 Application of microRNAs

MicroRNAs (miRNAs), as endogenous post-transcriptional regulators of gene expression, play critical roles in immune regulation and inflammatory responses, making them promising therapeutic targets for DN. Alterations in DN-associated miRNA expression can modulate the dysregulated renal immune microenvironment. Specifically, miR-192 and miR-21 are implicated in the development and progression of DN. They potentially influence disease outcomes by regulating inflammatory signaling pathways and modulating immune cell function, thereby offering novel avenues for therapeutic intervention (134).

Accumulating evidence has demonstrated that microRNA-218 (miR-218) is significantly downregulated in DN. miR-218 regulates the NF-κB signaling pathway-mediated inflammatory response by directly targeting and binding to inhibitor of nuclear factor kappa-B kinase subunit β (IKK-β), a key inflammation-associated molecule. This regulatory mechanism ultimately influences the progression of DN. Overexpression of miR-218 not only markedly attenuates renal tissue pathological damage but also effectively suppresses both local renal and systemic inflammatory responses. These findings indicate that miR-218 plays a pivotal role in modulating NF-κB-mediated inflammation, which serves as a central driver of DN progression (135, 136). These studies suggest that regulating the expression of specific miRNAs through gene therapy or pharmaceutical intervention may provide a novel therapeutic approach for DN.

### 6.4 Gut microbiota modulation

Targeting the gut microbiota and its metabolites has emerged as a promising and increasingly recognized therapeutic strategy for DN in recent years. Several approaches have been explored to modulate the gut microbiota. These include increasing dietary fiber intake to promote beneficial bacteria and enhance short-chain fatty acid (SCFA) production, as well as supplementing with probiotics or prebiotics to regulate microbial composition and improve intestinal barrier function. For instance, clinical evidence shows that Bifidobacterium supplementation significantly reduces serum levels of lipopolysaccharide (LPS) and trimethylamine (TMA), thereby alleviating renal inflammation and fibrosis. Another key approach is fecal microbiota transplantation (FMT) from healthy donors to restore gut microbiota homeostasis in DN patients. Preclinical studies have confirmed that FMT can effectively ameliorate gut microbiota dysbiosis in DN mouse models and mitigate renal injury. However, the clinical translation of FMT for DN still requires validation through large-scale, well-designed clinical trials. Collectively, these strategies help restore intestinal barrier integrity, reduce the production of harmful metabolites, and consequently alleviate systemic and renal inflammatory responses (115, 119, 120, 137).

### 6.5 Pharmaceutical therapy

Several pharmaceutical agents have shown promise in modulating the renal immune microenvironment in DN. Various drugs that target distinct signaling pathways exhibit unique mechanistic profiles. By modulating the activity of these pathways, therapeutic interventions can effectively slow the progression of diabetic nephropathy (**Table 2**). Growing evidence indicates that the combination of hydroxycaproic acid, canagliflozin, and valsartan exerts beneficial regulatory effects on the immune microenvironment in patients with DN. Specifically, this combination therapy has been shown to modulate levels of key inflammatory markers—such as high-sensitivity C-reactive protein (hs-CRP), IL-1, IL-6, and TNF-α—and to improve immune function in these individuals (138).

**Table 2:** Summary of major signaling pathways and targeted therapeutic interventions in diabetic nephropathy.

Signaling pathway	Primary activating factors in diabetic nephropathy	Downstream key effector molecules/cells	Induced renal pathological changes	Targeted intervention strategies	References
NF-κB	High glucose TLR4	NLRP3, Caspase-1, GSDMD, IL-1β, IL-18 GC, RETC	GMC proliferation with inflammatory cell infiltration RTEC dysfunction Renal interstitial fibrosis	Yitangkang: inhibit the TLR4/NF-κB/NLRP3 signaling pathway, pyroptosis and inflammatory responses, downregulate the activation of NF-κB,	(145)
	High glucose IL-1β, TNF-α, MCP-1 ROS, AGES	TNF-α, IL-1β, IL-6, MCP-1, IL-17, Mφ, NEU, GMC, RTEC	Glomerular injury Pc apoptosis and detachment TI injury Renal fibrosis	Baicalin:inhibit the NF-κB and MAPK pathway, activate of the Nrf2/HO-1 antioxidant pathway, alleviate oxidative stress and inflammation,	(144)
TGF-β/Smad	High glucose RAAS, ECM, ROS Inflammatory Cytokines	Smads GMC, RTEC, GEC, DC, Mφ	GBM thickening Mesangial expansion Glomerular endothelial injury Renal interstitial fibrosis	Pirfenidone: inhibit the TGF-β/Smad pathway, reduce ROS and pro-fibrotic cytokines	(63)
	High glucose	α-SMA, Collagen IV RETC, GEC, Mφ	Glomerular injury and sclerosis TI injury Renal interstitial fibrosis Increased Mφ & Lymp infiltration in TI	UC-MSCs: inhibit the expression and activation of TGF-β, and inflammatory responses	(41)
NLRP3Inflammasome	High glucose NETS	mIL-1β GEC, Pc	Loss or dysfunction of Pc Disruption of the glomerular filtration barrier, Structural damage to the glomeruli	PAD4 inhibitors: indirectly inhibit the activation of the NLRP3 inflammasome and reduce the formation of NETs.	(42)
JAK/STAT3	High glucose Angll IL-6	MCP-1, TGF-β, miR-34a, DNMT1 PC, MC, RTEC, RF, Mφ, Th17Cell, Treg	Glomerular injury TI injury Recruitment of Mφ and T cells to infiltrate renal tissue	IL-6 and its receptor inhibitors: inhibit of IL-6 or its receptor and indirectly block the activation of JAK/STAT3 JAK inhibitors: reduce proteinuria levels in DKD patients and alleviate renal inflammation	(71)
AMPK/SIRT1/NRF2	High glucose GLP-1 receptor agonist	IL-1β, TNF-α, IL-6, MCP-1, NF-κB, TGF-β1, Smad2/3, α-SMA, GPX4, SLC7A11, FSP1, FTH1, FPN1 GC, RETC	Glomerular Hypertrophy Vacuolar degeneration and shedding of RETC Collagen fiber deposition and TI fibrosis	Ferroptosis inhibitors: directly inhibit ferroptosis in renal tubular cells, alleviate kidney injury and fibrosis GLP-1 receptor agonists: activate the AMPK/SIRT1/NRF2 pathway, inhibit ferroptosis, inflammation, and fibrosis	(141)
PI3K/Akt	High glucose SHIP TGF-β	TGF-β1, α-SMA, CTGF, CoIII, TEC, GMC, RF	Promote EMT and enhance the fibrotic phenotype of cell MC proliferation Tubular injury Renal interstitial fibrosis	Specific PI3K/Akt pathway inhibitors: inhibit Akt phosphorylation and alleviate ECM accumulation Akt inhibitors: reduce the expression of TGF-β1 and α-SMA, alleviate ECM deposition in renal tubular cells	(80)

Sodium-glucose cotransporter 2 (SGLT2) is predominantly expressed in the renal tubules and mediates the reabsorption of 80%–90% of filtered glucose. Accumulating evidence indicates that sodium-glucose cotransporter 2 inhibitors (SGLT2i) confer renal protective effects that extend beyond glucose lowering. They provide comprehensive protection in DN through multifaceted modulation of renal physiological functions, particularly by targeting key pathological mechanisms such as improving renal hemodynamics and ameliorating maladaptive renal phenotypes. Specifically, SGLT2i directly modulate sodium reabsorption in the proximal tubules, restoring tubuloglomerular feedback (TGF) balance and thereby fundamentally improving intraglomerular pressure regulation. Glomerular hyperfiltration is a hallmark of early-stage DN and a critical contributor to progressive nephron injury. By correcting this hemodynamic abnormality, SGLT2i effectively reduce glomerular hyperfiltration, mitigate intraglomerular hypertension, and delay the progression of renal damage. Furthermore, SGLT2i indirectly attenuate renal injury by improving systemic metabolic and circulatory homeostasis, thus exerting synergistic renoprotective effects (139).

Semaglutide exerts multifaceted renoprotective effects through several interrelated mechanisms. It modulates renal-related gene expression, suppresses macrophage infiltration and inflammatory cytokine release, and alleviates tubulointerstitial inflammation. Furthermore, it preserves podocyte integrity and glomerular filtration barrier function to reduce proteinuria. Additionally, semaglutide regulates genes involved in extracellular matrix remodeling, thereby inhibiting glomerulosclerosis and fibrosis. Additionally, semaglutide improves insulin sensitivity, modulates glucose metabolism, and lowers blood glucose levels, contributing to synergistic renal protective effects in DN (140). The renoprotective action of semaglutide follows an upstream activation–midstream regulation–downstream effect cascade. Specifically, semaglutide binds to the glucagon-like peptide-1 receptor (GLP-1R), activating the cAMP/PKA/CREB signaling pathway and upregulating the expression of β-klotho (KLB). KLB acts as a central hub that links upstream GLP-1R signaling to downstream metabolic regulation by interacting with specific kinases to activate AMP-activated protein kinase (AMPK). Activation of the AMPK/SIRT1/NRF2 axis modulates antioxidant responses and iron homeostasis, thereby suppressing ferroptosis—an effect that represents the core effector mechanism through which semaglutide exerts its anti-ferroptotic action. Upon AMPK activation, ferroptosis is inhibited via coordinated regulation of three key processes—lipid metabolism, antioxidant defense, and iron metabolism—while inflammatory and fibrotic pathways are concurrently suppressed, ultimately providing renal protection in DN (141).

The mineralocorticoid receptor (MR) belongs to the steroid hormone intracellular receptor family and is widely expressed in tissues and organs such as the kidneys and heart. Its ligands and functional activities exhibit tissue specificity, providing a mechanistic basis for the multi-system impacts of MR overactivation. DN patients frequently present with MR overactivation: this process not only regulates renal sodium and water reabsorption via the classical pathway, but also induces renal oxidative stress, inflammatory responses, and fibrosis through non-classical pathways, thereby accelerating glomerular injury and renal function deterioration. Non-steroidal mineralocorticoid receptor antagonists (MRAs) can block the pathological effects of MR overactivation, thus delaying the progression of DN (142).

Numerous traditional Chinese medicine (TCM) components have demonstrated therapeutic potential in DN. Growing evidence indicates that Angelica sinensis polysaccharide (ASP) attenuates renal inflammation and fibrosis by downregulating the mRNA expression of key inflammatory mediators, such as MCP-1, TNF-α, and IL-1β, in renal tissues (143). Baicalin can simultaneously activate the Nrf2/HO-1 antioxidant signaling pathway, upregulate the expression of HO-1 and NQO-1, suppress the MAPK-mediated inflammatory pathway, and downregulate the phosphorylation of Erk1/2, JNK, and p38, thereby alleviating oxidative stress and inflammation (144). Accumulating evidence demonstrates that the TCM compound Yitangkang inhibits pyroptosis and inflammatory responses in renal tissue cells of DN mice. Its mechanism involves modulating the TLR4/NF-κB/NLRP3 inflammasome signaling pathway. Specifically, Yitangkang activates TLR4-mediated signaling to regulate the NF-κB pathway, suppresses NLRP3 inflammasome protein expression in renal tissues, and consequently reduces the levels of cleaved GSDMD-N, cleaved caspase-1, IL-1β, and IL-18, thereby attenuating renal inflammatory cell infiltration (145). In a hyperglycemic microenvironment, glomerular endothelial cells, renal tubular epithelial cells, and other intrinsic renal cells are activated and secrete substantial amounts of exosomes enriched with microRNA-21 (miR-21). These exosomes diffuse through the renal tissue microenvironment to reach podocytes, where they deliver miR-21 via membrane fusion, resulting in abnormally elevated intracellular levels of miR-21 in podocytes. Accumulating evidence indicates that astragaloside IV (AS-IV) modulates the miRNA biosynthesis pathway in glomerular endothelial cells, downregulating miR-21 transcription and reducing its production. Furthermore, AS-IV inhibits the biogenesis and secretion of exosomes, thereby decreasing the release of miR-21-enriched exosomes into the renal microenvironment. This effectively interrupts the intercellular transmission of miR-21 to podocytes, preventing pathological accumulation of miR-21 in these cells during DN (146). Artemether, a derivative of artemisinin, has demonstrated significant therapeutic potential in metabolic disorder-related diseases. Its multifaceted hypoglycemic and anti-inflammatory immunomodulatory effects offer a promising new avenue for disease intervention. The AMPK/mTOR signaling pathway, a central regulator of energy metabolism and cell proliferation, is closely implicated in metabolic homeostasis and inflammatory responses, positioning it as a key molecular target for artemether’s action. By modulating this pathway, artemether effectively down-regulates the expression of pro-inflammatory cytokines, alleviates their disruption of the insulin signaling cascade, and thereby achieves a synergistic effect combining glucose-lowering and anti-inflammatory activities (147). Artesunate inhibits the mRNA expression of key inflammatory cytokines, including IL-1β, IL-6, and TNF-α, while simultaneously down-regulating core proteins in inflammatory signaling pathways such as inducible nitric oxide synthase (iNOS) and NF-κB, thereby suppressing aberrant inflammatory responses in renal tissues (148). TCM primarily follows the therapeutic principles of clearing heat, promoting fluid production, tonifying qi, nourishing yin, and activating blood circulation to eliminate turbidity. It has demonstrated favorable clinical efficacy in the treatment of DN and shows promising developmental potential. Therefore, investigating effective TCM-based interventions for DN carries substantial clinical and scientific significance.

**Table 1** illustrates that activation of specific signaling pathways in diabetic nephropathy can lead to a range of renal pathological alterations, including glomerular injury, mesangial expansion, tubulointerstitial fibrosis, and podocyte dysfunction. Targeted intervention strategies directed at distinct signaling pathways exhibit unique mechanistic characteristics. By modulating the activity of these pathways, such interventions can effectively mitigate the progression of diabetic nephropathy.

## 7 Future outlook

### 7.1 Refined research on immune cell subsets

Currently, the understanding of renal immune cells largely relies on traditional phenotypic classification. However, advances in scRNA-seq, spatial transcriptomics, and related technologies have introduced powerful tools for dissecting immune cell heterogeneity. These approaches enable the identification of previously unrecognized cell subsets, enhance insights into intercellular crosstalk, and reveal dynamic alterations in the immune microenvironment across distinct pathological stages of DN. The application of scRNA-seq and complementary methods allows researchers to uncover novel immune cell subpopulations, including functionally distinct macrophage and T-cell subtypes. These techniques further enable the characterization of their spatial distribution, functional roles, and regulatory mechanisms across disease progression (123, 124, 149). This will offer a more comprehensive perspective on the complex mechanisms underlying diabetic nephropathy, thereby establishing a crucial foundation for future mechanistic studies and the development of effective intervention strategies.

### 7.2 Immune microenvironment signaling networks and cell-cell interactions

In-depth investigations into the renal immune microenvironment have revealed intricate interconnections among multiple factors. These include inflammation, oxidative stress, cell death, cellular senescence, dysregulated lipid metabolism, and gut microbiota-derived metabolites. These interactions collectively form a coordinated regulatory network that modulates immune microenvironment homeostasis and directly contributes to the initiation and progression of renal injury (3, 66, 90, 110, 111). Future research should therefore focus on elucidating the molecular mechanisms through which these factors interact to drive diabetic nephropathy progression. This will provide a crucial theoretical foundation for understanding pathogenesis and developing targeted therapies.

### 7.3 Advancing research on immune microenvironment biomarkers

Currently identified immune microenvironment-related biomarkers remain largely confined to preclinical research and lack validation through large-scale, multi-center clinical studies. Future efforts should focus on rigorous clinical evaluations to confirm the utility of biomarkers such as FSTL1, CX3CR1, and Cit-H3 in early diagnosis, prognosis assessment, and therapeutic monitoring. Concurrently, the development of efficient and non-invasive detection technologies is essential to support their clinical translation. Moreover, integrating multiple biomarkers into composite panels may significantly enhance diagnostic and prognostic accuracy, representing a promising direction for future research (124, 149).

### 7.4 In-depth research on gut microbiota

The role of the gut microbiota in DN has garnered increasing attention, but the mechanisms by which it modulates the renal immune microenvironment via metabolites remain incompletely elucidated. In the future, it is necessary to clarify the interaction mechanisms between specific gut microbiota and their metabolites and renal immune cells such as macrophages and T cells, and dissect the regulatory axis of “gut microbiota – metabolites – immune cells – renal injury”. Meanwhile, clinical studies should be conducted on precision probiotic supplementation and gut microbiota-based FMT intervention strategies to verify their safety and efficacy in DN. Additionally, exploring the cross-organ regulatory mechanisms of the gut-kidney immune axis mediated by the gut microbiota can provide novel insights for DN intervention.

### 7.5 Clinical translation of precision targeted therapy

In the future, individualized therapeutic strategies should be developed based on comprehensive immune microenvironment profiling in patients with diabetic nephropathy DN, including immune cell subset distribution, inflammatory cytokine levels, and gut microbiota composition, to enable precise targeted interventions. A critical research priority also lies in advancing novel drug delivery systems that enhance renal drug accumulation and minimize systemic side effects. Furthermore, exploring combination therapies represents a highly promising avenue for clinical translation. Such therapies would simultaneously target immune cells, modulate gut microbiota, and inhibit key inflammatory signaling pathways, thereby achieving multidimensional regulation of the renal immune microenvironment.

## 8 The limitations of this review

### 8.1 Incomplete understanding of immune mechanisms and the gut-kidney axis

The review on immune cells and their underlying mechanisms remains insufficient, and the molecular network mediating interactions among various immune cells has not yet been fully elucidated, which hinders a comprehensive understanding of how the immune microenvironment influences the progression of diabetic nephropathy. Furthermore, the dynamic alterations in the immune microenvironment across different pathological stages of diabetic nephropathy have not been systematically characterized, and the mechanistic interactions between immune cells and intrinsic renal cells remain poorly understood. In addition, the role of gut microbiota requires further investigation. The specific molecular pathways through which distinct microbial species and their metabolites modulate the renal immune microenvironment are still not well defined.

### 8.2 Insufficient clinical translation of biomarkers and immunomodulatory therapies

On one hand, the inadequacy of current biomarkers is evident in the limited number listed, most of which remain at the stage of basic research or small-scale clinical exploration. A lack of multi-center, large-sample, and long-term follow-up studies has hindered the validation of their performance, leaving the sensitivity and specificity of these biomarkers in diagnosing diabetic nephropathy, as well as their utility in prognostic assessment, insufficiently established. On the other hand, the clinical evidence supporting the proposed treatment strategies remains weak. Interventions targeting the immune microenvironment—such as modulation of macrophage polarization, expansion of Tregs, and fecal microbiota transplantation—are still confined to preclinical animal models or early-phase clinical trials.

### 8.3 Lack of systematic research on traditional chinese medicine and integrated pathological networks

Although the present study discusses the effects of traditional Chinese medicine components such as Angelica sinensis polysaccharides and *Scutellaria baicalensis*, it primarily focuses on isolated constituents or specific cell signaling pathways, thereby lacking a systematic investigation into how traditional Chinese medicine formulas regulate the immune microenvironment. Furthermore, there is insufficient elaboration on the interactions between the immune microenvironment and key pathological mechanisms—including oxidative stress, dysregulated lipid metabolism, and programmed cell death—limiting the construction of an integrated pathological network and constraining a comprehensive understanding of the disease’s complex mechanisms.

## 9 Conclusion

In conclusion, the renal immune microenvironment plays a central role in the initiation and progression of DN. Metabolic disturbances, particularly hyperglycemia, induce profound alterations in this microenvironment, including infiltration and activation of diverse immune cell populations, elevated production of pro-inflammatory cytokines and chemokines, and dysregulated activation of inflammatory signaling pathways. These immune changes dynamically interact with intrinsic renal non-immune cell injury, extracellular matrix accumulation, and systemic influences from gut microbiota dysbiosis and their metabolites. This interplay collectively forms a complex pathological network that drives renal inflammation, fibrosis, and functional decline. A deeper understanding of the cellular composition and regulatory mechanisms within the renal immune microenvironment not only enhances our comprehension of DN pathophysiology but also establishes a crucial basis for identifying novel biomarkers and developing targeted therapeutic interventions. Future studies should harness advanced technologies to systematically unravel the intricacies of this microenvironment and accelerate the translation of mechanistic insights into clinical applications. Guided by the principles of precision medicine, individualized treatment strategies should be tailored to the unique immune profiles of patients, paving the way for more effective diagnosis and management of DN.

## Glossary

SLC7A11: Solute Carrier Family 7 Member 11
CTGF: Connective Tissue Growth Factor
PAD4: Peptidylarginine Deiminase 4
GPX4: Glutathione Peroxidase 4
FPN1: Ferroportin 1
Collagen IV: Collagen Type IV
NEU: Neutrophil
Mφ: Macrophage
NETs: Neutrophil Extracellular Traps
GEC: Glomerular Endothelial Cell
ESRD: End-Stage Renal Disease
SCFAs: Short-Chain Fatty Acids
PDGF: Platelet-Derived Growth Factor
ICAM-1: Intercellular Adhesion Molecule-1
FGF: Fibroblast Growth Factor
CTLs: Cytotoxic T Lymphocytes
Cit-H3: Citrullinated Histone H3
LXA4: Lipoxin A4
VEGF: Vascular Endothelial Growth Factor
CSF-1: Colony-Stimulating Factor 1
PAMPs: Pathogen-Associated Molecular Patterns
NETs: Neutrophil Extracellular Traps: NETs are reticular structures released by activated neutrophils. They are composed of decondensed chromatin, histones, and antimicrobial granule proteins, enabling them to capture and kill pathogens. While essential for anti-infective immunity, excessive NET formation is closely associated with the pathogenesis of autoimmune diseases, thrombosis, and organ damage
PAMPs: Pathogen-Associated Molecular Patterns: PAMPs are conserved molecular structures derived from microorganisms, including bacterial lipopolysaccharide (LPS), viral double-stranded RNA (dsRNA), and fungal cell wall components. As non-self motifs, they are specifically recognized by host pattern recognition receptors (PRRs), triggering critical signaling cascades that initiate anti-infective immune defenses. DAMPs, Damage-Associated Molecular Patterns: DAMPs are endogenous molecules released from damaged or dying cells that are recognized by the innate immune system. Their recognition activates innate immune responses and promotes inflammatory processes
UC-MSCs: Human Umbilical Cord Mesenchymal Stem Cells
SHIP: Src Homology 2 Domain-Containing Inositol Phosphatase
CoIII: Collagen Type III
FSP1: Ferroptosis Suppressor Protein 1
miR-34a: MicroRNA-34a
GMC: Glomerular Mesangial Cell
Pc: Podocyte
DC: Dendritic Cell
GC: Glomerular Cell
GBM: Glomerular Basement Membrane
TEC: Tubular Epithelial Cells
TGF-β: Transforming Growth Factor-β
TMAO: Trimethylamine N-oxide
ROS: Reactive Oxygen Species
VCAM-1: Vascular Cell Adhesion Molecule-1
IFN-γ: Interferon-γ
APCs: Professional Antigen-Presenting Cells
MMPs: Matrix Metalloproteinases
MSCs: Mesenchymal Stem Cells
HIF-1α: Hypoxia-Inducible Factor-1α
FMT: Fecal Microbiota Transplantation
DAMPs: Damage-Associated Molecular Patterns
EMT: Epithelial-Mesenchymal Transition
mIL-1β: Mature Interleukin-1 beta
TLR4: Toll-like receptor 4
FTH1: Ferritin Heavy Chain 1
DNMT1: DNA Methyltransferase 1
RTEC: Renal Tubular Epithelial Cell
TI: Tubulointerstitial
RF: Renal Fibroblast
ECM: Extracellular Matrix
MC: Mesangial Cell
Lymp: Lymphocyte
AGEs: Advanced Glycation End Products
LPS: Lipopolysaccharide
MCP-1: Monocyte Chemoattractant Protein-1
NO: Nitric Oxide
MHC: Major Histocompatibility Complex
NF-κB: Nuclear Factor-κB
TIMPs: Tissue Inhibitors of Metalloproteinases
DAMPs: Damage-Associated Molecular Patterns
Tim-3: T-cell Immunoglobulin and Mucin Domain-Containing Molecule 3
MAPK: Mitogen-Activated Protein Kinase
NLRP3 inflammasome: NLR Family Pyrin Domain-Containing 3
